# Global research trends on precision cancer medicine-related rashes (2008-2021): A bibliographic study

**DOI:** 10.3389/fimmu.2022.1002034

**Published:** 2022-08-26

**Authors:** Fangmin Zhao, Rui Yu, Shuyi Chen, Shuya Zhao, Lin Sun, Zeting Xu, Yao Zhang, Shuying Dai, Gaochenxi Zhang, Qijin Shu

**Affiliations:** ^1^ Department of First Clinical Medical College, Zhejiang Chinese Medical University, Hangzhou, China; ^2^ Department of Oncology, The First Affiliated Hospital of Zhejiang Chinese Medical University, Hangzhou, China

**Keywords:** precision cancer medicine, targeted therapy, checkpoint inhibitors, rash, bibliometric analysis

## Abstract

**Background:**

Precision cancer medicine-related rashes are a kind of skin and mucous lesions caused by precision therapy. More and more evidences indicated that such events should not be ignored in the course of anti-tumor therapy. Since cancer treatment entered the “Precision Era”, there has been a rapid increase in this field. However, there was few bibliometric studies to provide an overall review of this field. This study aims to evaluate the literature output and trends in researches on precision cancer medicine-related rashes from a global perspective.

**Methods:**

Collected publications on precision cancer medicine-related rashes from the Web of Science Core Collection database, which were limited to articles and reviews in English. Microsoft Excel, VOS viewer and CiteSpace V were used for quantitative and visual analysis.

**Results:**

A total of 1,229 papers were identified. From 2008 to 2021, annual publications increased year by year. The United States published the most papers in this field (44.9%) and ranking first in citation frequency (19,854 times) and H-index (69). The University of Texas system ranks first with 98 papers published. Lacouture M.E and Robert C were the principal investigators. Cancers has the largest number of articles published, with 70 articles. In recent years, there have been research hotspots related to immunotherapy, including ipilimumab, immunotherapy, tumor microenvironment, association, checkpoint inhibitor, and cutaneous adverse event.

**Conclusion:**

Precision cancer medicine-related rashes are a hot research topic in oncology. The number of relevant publications will increase dramatically. “Checkpoint inhibitors”, “skin adverse events”, “associations” and “tumor microenvironment” may become research hotspots in the future.

## Introduction

The field of precision cancer medicine is constantly broadening with the biotechnological breakthroughs. It brings safer and more novel therapies to patients, such as gene-targeted therapy, immune-targeted approaches, etc. (1). Clinical trials have shown that patients receiving targeted therapy matched to their molecular changes had better response, time to treatment failure and survival than patients receiving unmatched therapy, and progression-free survival could be improved by approximately 30% (2). Immunotherapy, including checkpoint blockade, personalized vaccines, etc, demonstrated a survival benefit versus chemotherapy, exhibiting a five-fold increase in 5 years’ overall survival (OS) rate (13.4% vs 2.6%) (3, 4).

However, these precision therapies also generate various toxicities, mainly involving the skin, gut, liver, lung, and endocrine glands, but can potentially affect any tissue (5, 6). Rashes are one of the most common adverse reactions, which often profoundly reduces patients’ quality of life (7–9), affects the treatment outcomes. It has become a major challenge in accelerating the implementation of precision medicine. Over the past few years, the accumulating evidence has shown that the occurrence of rashes may herald better efficacy of precision therapy. The pathogenesis, diagnosis, prevention and treatment of precision cancer medicine-related rashes have received considerable attention, and many scholars have published relevant research articles. To our knowledge, few systematic investigations have been conducted on the scientific output and current status of research on precision cancer medicine-related rashes from a global perspective.

In this study, we performed a bibliometric analysis to systematically evaluate studies of precision cancer medicine-related rashes. We combine statistical methods with data visualization to analyze the bibliography of relevant literature to identify global research trends and hotspots in this field.

## Materials and methods

### Data retrieval and literature screening

**Figure 1:**
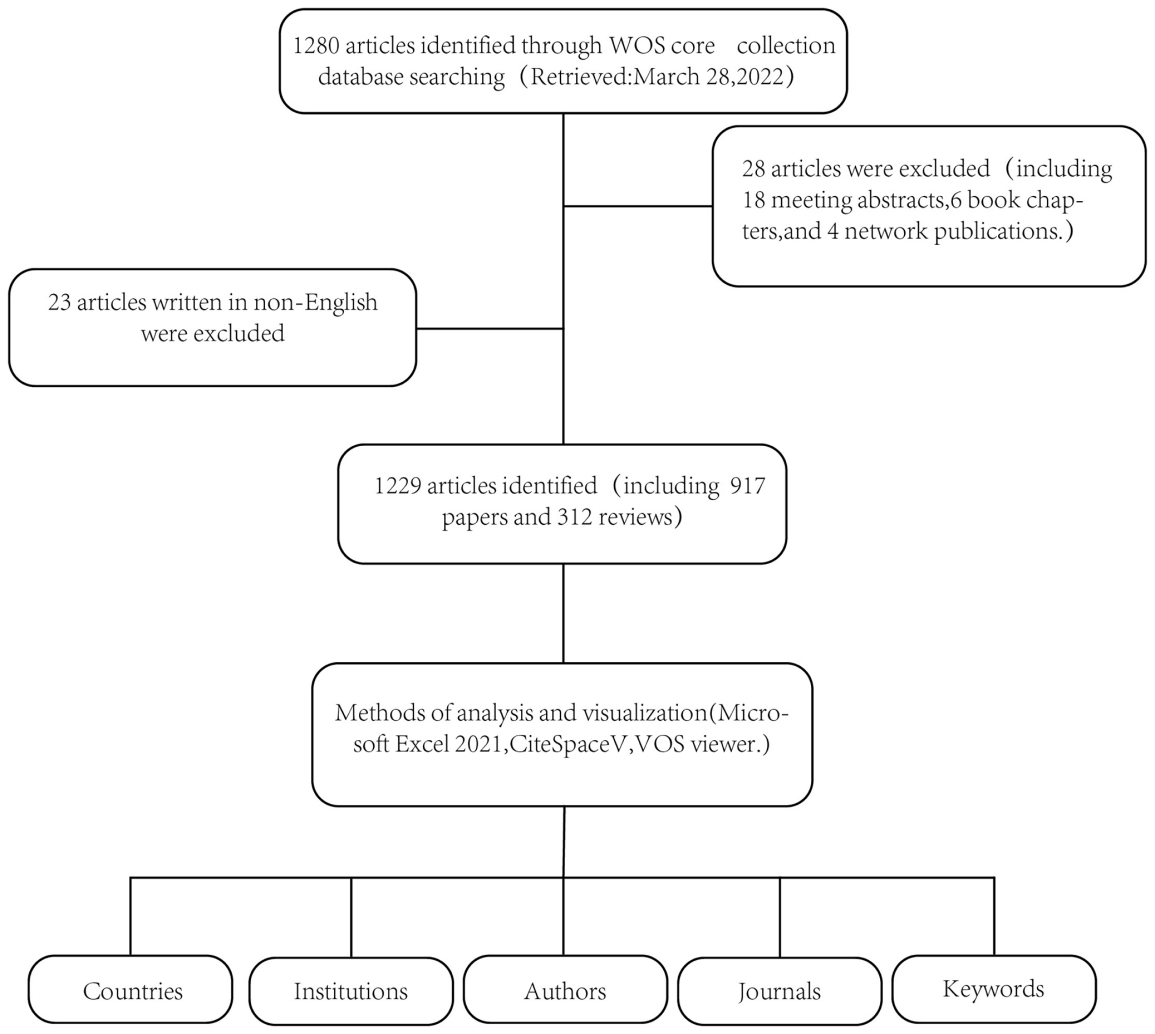
Flowchart for the selection of publications included in this study.

In this study, the Science Citation Index (SCI) Expanded of Web of Science Core Collection (WoSCC) database was chosen as the data source. Searches were conducted using the following search strategy: (“Immunotherapies” or “Molecular Targeted Therapies” or “Targeted Therapy” or “Molecular Targeted” or “Targeted Molecular Therapy” or “Molecular Therapy” or “Targeted” or “Targeted Molecular”) and (“Skin Rash” or “Rash” or “Skin” or “Exanthem”). The time is from the inception of the database to March 28, 2022. Language was restricted to English. For manuscript types, we selected original articles and reviews, and excluded all other source types to ensure quality research (**Figure 1**).

### Data extraction and analysis

The data were extracted independently by two authors, including annual study, countries, institutions, authors, journals, citations, and keywords. We used Microsoft Excel 2021 for quantitative analysis to calculate the total number of annual publications and the average citations of per publication, the number of annual publications and cumulative number over the years in countries, and the cumulative number of papers published by institutions, authors and journals. As evaluation metrics for publications, we mainly used Impact Factor (IF) and category data published by Journal Citation Reports (JCR) in 2021 to assess the quality of scientific information. The H-index (10) was also used to assess researchers’ scientific output and academic standing, as well as the productivity and influence of countries, institutions, and journals. The H-index means that if a researcher’s H-index is h, then the researcher has published at least h papers, and each paper has been cited at least h times.

For the visualized analyses, VOS viewer (version5.8 R3) was used for co-authorship and co-citation analyses of countries, institutions, authors, journals, and co-occurrence analyses of keywords. CiteSpace V (version5.8 R3) was used to create the dual map overlay of journals, and generate a timeline view of keywords. Each node in the graph represents different parameters including countries, institutions, keywords, etc. The weighting of parameters determined the size of the nodes, such as the number of publications, the number of citations, or the frequency of occurrence. The higher the weight, the larger the node. Nodes and lines are colored by the cluster they belong to. Lines between nodes represent links. The Total Link Strength (TLS) indicates the total co-authorship and co-citation link strength between countries, institutions and authors.

## Research ethics

Ethical approval was not required in our study, as the data used in this study were downloaded from public databases and did not involve any new studies in humans or animals.

## Results

### Publication outputs and citation trend

**Figure 2:**
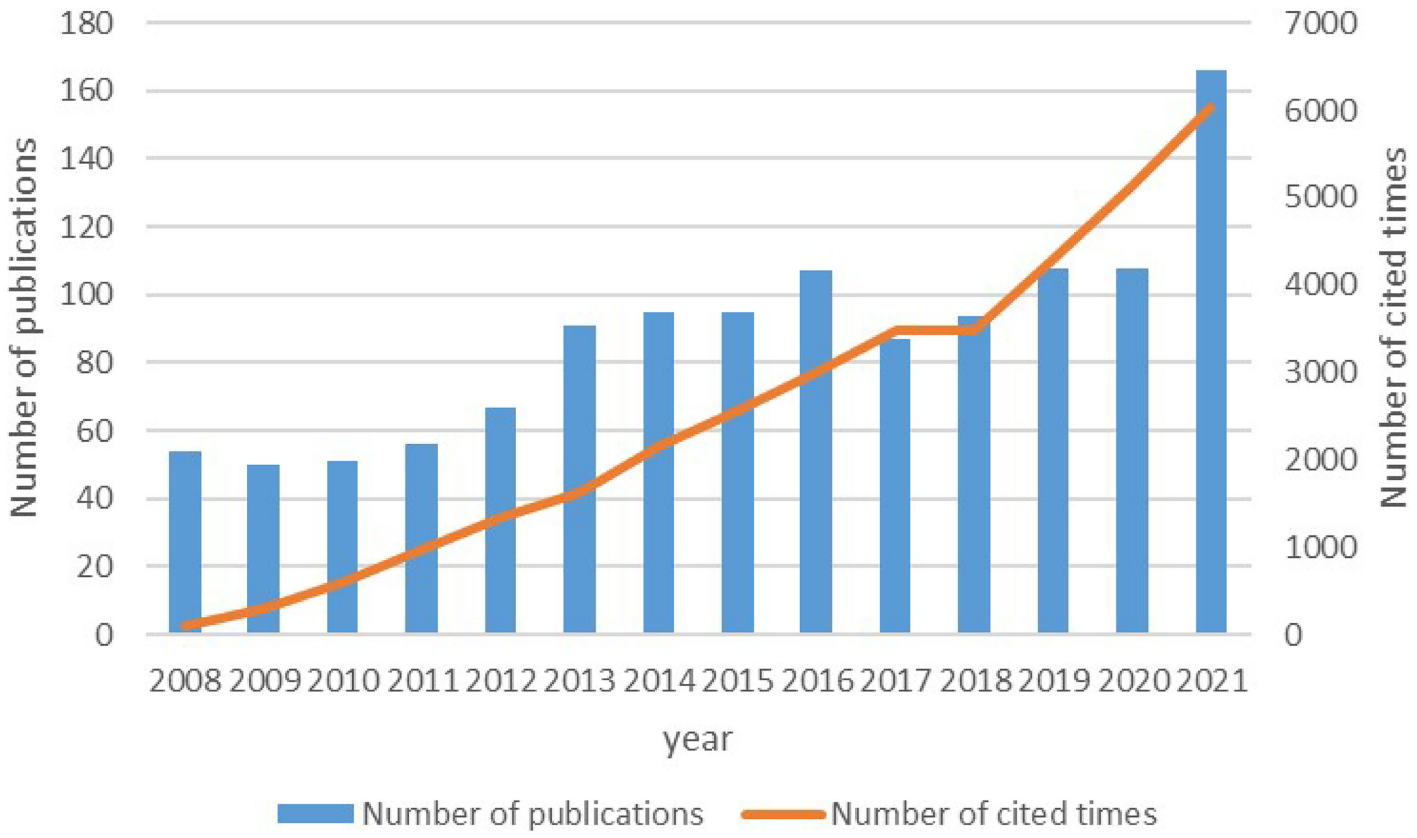
Trend of the number of articles published annually and the total amount of citations of annual articles.

A total of 1,229 articles on precision cancer medicine-related rashes including 917 papers and 312 reviews were identified from WOSCC database on March 28,2022. The number of publications from 2008 to 2021 has been increasing, especially in the past two years significantly (**Figure 2**). According to the search results, the sum number of citations was 31,569, and the average number of citations was 25.69. In addition, the total citation frequency of the included articles was 35,101 times, and the average citation frequency of the articles was 28.56 times. H-index of the academic field was 83 during this time period, indicating that the academic output in this field had research value and prospect.

### Distribution of countries

**Figure 3:**
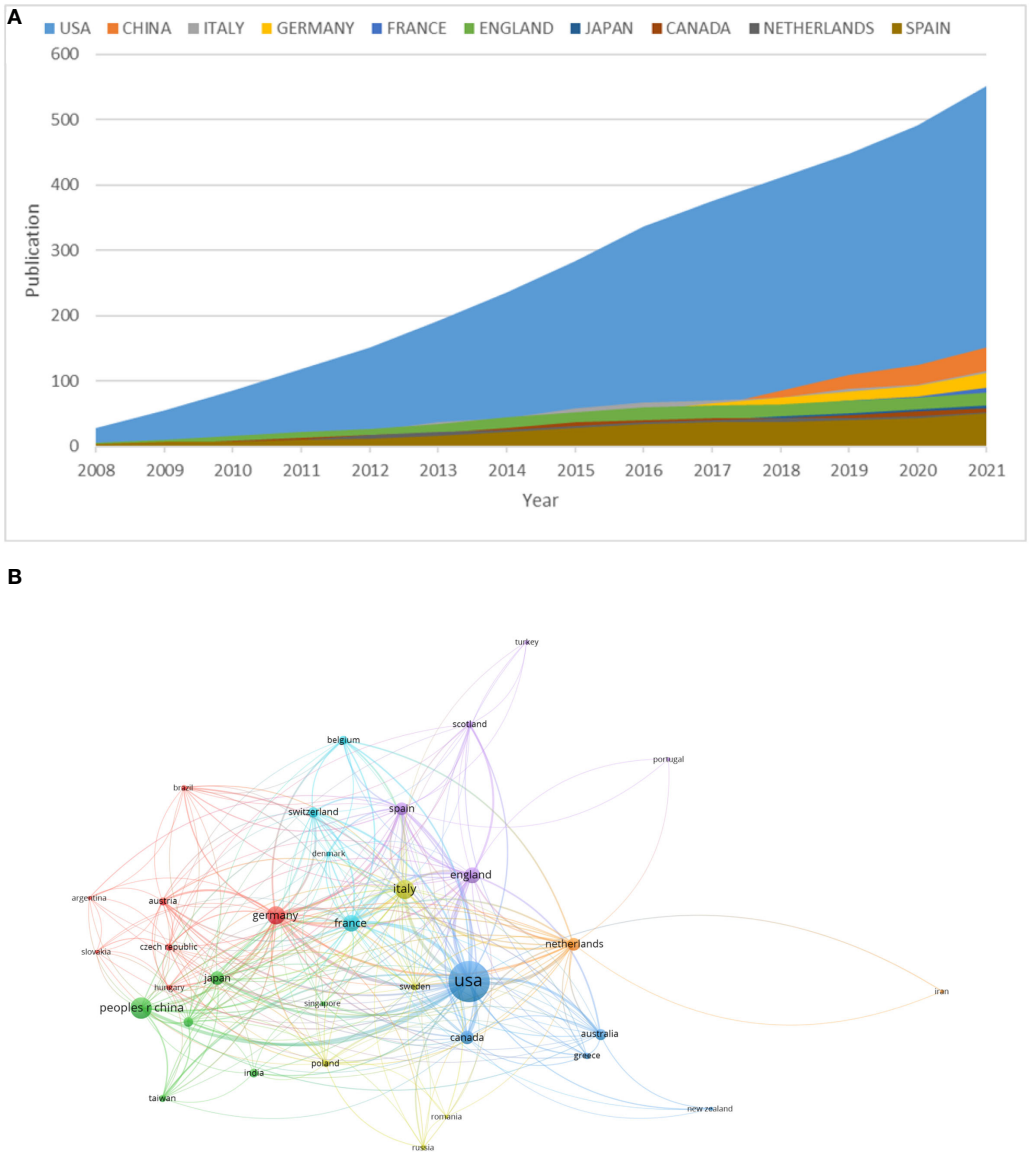
**(A)** The changing trend of the annual publication counts in the top 10 countries. **(B)** The country citation network visualization map generated by VOS viewer (version5.8 R3). USA, The United States of America.

**Table 1** lists the top 10 countries with the most articles that associated with precision cancer medicine-related rashes, and **Figure 3A** shows changing trend of the number of relevant publications in these countries from 2008 to 2021. The United States had the largest number of publications, accounting for 44.9% of the total number of articles (552/1,229), followed by China (12.4%, 152/1,229) and Italy (9.4%, 116/1,229). The publications from the United States were cited the most (19,854 times) and had the highest H-index (69). The total number of articles overlapped because of cooperation between countries. **Figure 3B** shows the literature citation relationship between countries. The largest TLS of country was United States (TLS=322), followed by German (TLS=167) and France (TLS=136).

**Table 1:** The top 10 productive countries with publications.

Rank	Countries	Article count	Percentage (n/1229)	H-index	TLS	Total citations	Average citation per article
1	United States	552	44.90%	69	322	19,854	35.97
2	China	152	12.40%	26	80	2,930	19.28
3	Italy	116	9.40%	31	130	4,578	39.47
4	Germany	113	9.20%	32	167	4,242	37.54
5	France	90	7.30%	34	136	5,954	66.16
6	England	82	6.70%	31	131	4,390	53.54
7	Japan	63	5.10%	23	53	2,413	38.2
8	Canada	59	4.80%	28	81	2,160	36.61
9	Netherlands	52	4.20%	26	105	2,314	44.5
10	Spain	51	4.10%	23	109	2,000	39.22

### Distribution of institutions

A total of 2,057 institutions published articles in precision cancer medicine-related rashes. The collaboration between institutions had allowed more agencies to participate in this area. The top 10 institutions ranked by the number of articles are showed in **Table 2**. The vast majority of institutions were from the United States. University of Texas system contributed the most publications, followed by UTMD Anderson Cancer Center and Harvard University. University of Texas system had the highest H-index, and Unicancer from France had the most average citation per article.

**Table 2:** The top 10 productive institutions ranked by the numbers of publications.

Rank	Institutions	Countries	Article count	H-index	Total citations	Average citation per article
1	University of Texas system	United States	98	36	3,551	36.23
2	UTMD Anderson Cancer Center	United States	84	33	2,975	35.42
3	Harvard University	United States	70	26	3,515	50.21
4	University Of California System	United States	54	26	2,009	37.2
5	Unicancer	France	48	27	4,603	95.9
6	Memorial Sloan Kettering Cancer Center	United States	47	23	1,857	39.51
7	National Institutes of Health (NIH)	United States	42	23	2,006	47.76
8	Dana Farber Cancer Institute	United States	36	18	1,715	47.64
9	National Cancer Institute (NCI)	United States	35	21	1,808	51.66
10	Inserm	France	32	15	3,119	97.47

**Figure 4:**
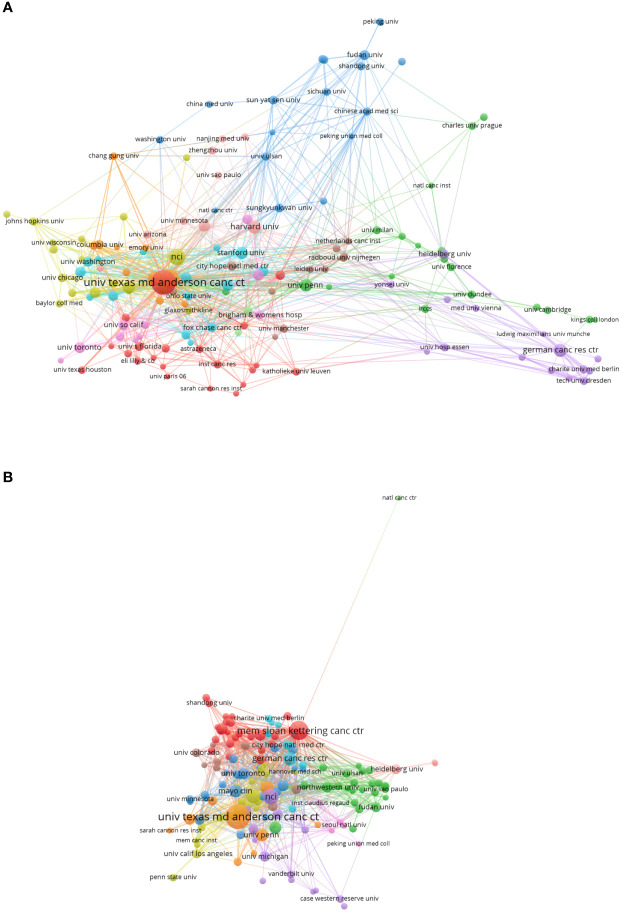
**(A)** The institutions’ collaboration network visualization map generated by VOS viewer (version5.8 R3) **(B)** The institutions’ citation network visualization map generated by VOS viewer (version5.8 R3).

**Figures 4A, B** show the institutions’ collaboration and citation network visualization map generated by VOS viewer (version5.8 R3). As shown in the **Figure 4A**, the map had 150 items and 967 links. The 150 items were grouped into 11clusters based on color. It meant that the institutions in the same cluster were closed related. The network map of citation analysis in **Figure 4B** presented 144 items and 1,048 links. The top institution with largest TLS was Memorial Sloan Kettering Cancer Center (TLS=204), and all other institutions had less than 100 TLS.

B. The institutions’ citation network visualization map generated by VOS viewer (version5.8 R3).

### Authors and co-cited authors

A total of 8,422 authors appeared in the 1,229 articles. The top 10 most productive authors in the publications were listed in **Table 3**. Lacouture M.E from United States contributed the most articles (18 articles) and the highest H-index (13). Robert C from Canada had the most average citation per articles (188.55 times per articles).

**Table 3:** The top 10 most productive authors in publications.

Rank	Author	Article count	H-index	Countries	Total citations	Average citation per article	Institutions
1	Lacouture ME	18	13	United States	491	27.28	Memorial Sloan-Kettering Cancer Center
2	Robert C	11	9	Canada	2,074	188.55	Minist Environm & Lutte Changements Climat
3	DiGiovanni J	11	8	United States	347	31.55	University of Texas Austin
4	Wang Y	9	5	China	165	18.33	Chinese people’s liberation army general hospital
5	Li J	8	5	China	523	63.38	Fudan University
6	Zhang Q	8	5	China	141	17.63	Nanjing Medical University
7	Ascierto PA	7	4	Italy	840	120	IRCCS Fondazione Pascale
8	Belum VR	7	7	India	122	17.43	ICRISAT
9	Dummer R	7	6	Switzerland	636	90.86	University of Zurich
10	Loquai C	7	3	Germany	62	8.86	Johannes Gutenberg University of Mainz

**Figure 5:**
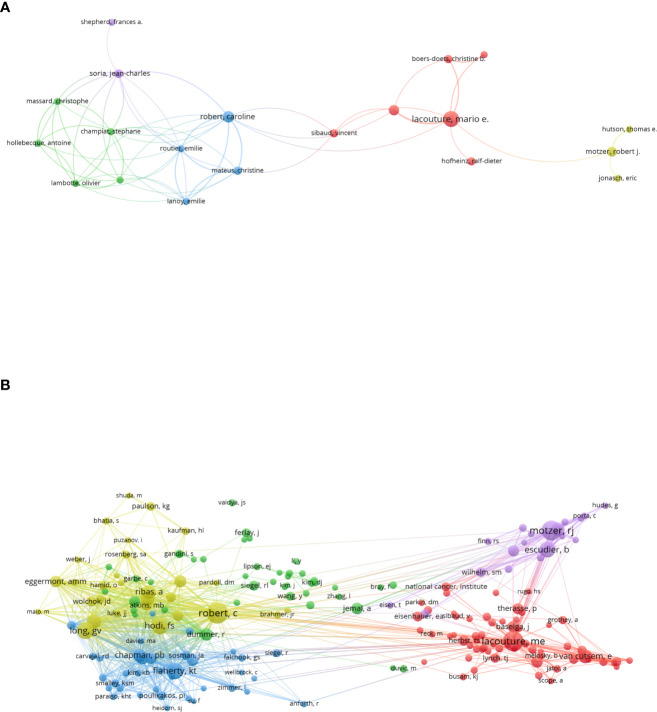
**(A)** The authors’ collaboration network visualization map generated by VOS viewer (version5.8 R3). **(B)** The authors’ co-citation network visualization map generated by VOS viewer (version5.8 R3).

**Figure 5A** illustrated the collaboration between the authors. Prolific authors like Lacouture M.E and Robert C had the active network of collaborators. The authors’ co-citation network included 188 items, 5 clusters and 7,936 links (**Figure 5B**). The top three authors with largest TLS were Robert C(TLS=5,187), Long G.V(TLS=3,586) and Hodi F.S(TLS=3,286).

### Journals and co-cited journals

A total of 204 journals published articles on precision cancer medicine-related rashes. The top 10 journals were listed based on a comprehensive quality assessment (**Table 4**). As shown in the table, 299 articles were published in the top 10 journals, accounting for 24.3% of the included articles. Cancers (IF 2021 = 6.639) came out the most articles (count:70 pieces), the following were Frontiers in Oncology (IF 2021 = 6.244) and Oncotarget (this journal was not in the latest JCR). Of the top 10 journals, five were from the United States, three from England and two from Switzerland. Clinical Cancer Research and Cancer Research had the highest H-index (22). Meanwhile Clinical Cancer Research had the most total citations (1,709 times) and highest IF (IF 2021 = 12.531).

**Table 4:** The top 10 journals of research ranked by publication number.

Rank	Journal Title	Country	Count	IF (2021)	Quartile in category (2021)	H-index	Total citations
1	Cancers	Switzerland	70	6.639	Q2	11	395
2	*Frontiers in Oncology*	Switzerland	32	6.244	Q3	9	700
3	*Oncotarget*	United States	32	/	/	14	460
4	*Clinical Cancer Research*	United States	27	12.531	Q1	22	1,709
5	*Melanoma Research*	United States	27	3.599	Q2/3	10	374
6	*Cancer Researc*h	United States	26	12.701	Q1	22	1,668
7	*Investigational New Drugs*	United States	25	3.85	Q2/3	13	712
8	*Oncogene*	England	21	9.867	Q1	13	701
9	*British Journal of Cancer*	England	20	7.64	Q1	14	701
10	*European Journal of Cancer*	England	19	9.162	Q1	12	1,502

IF, Impact factor.

**Figure 6:**
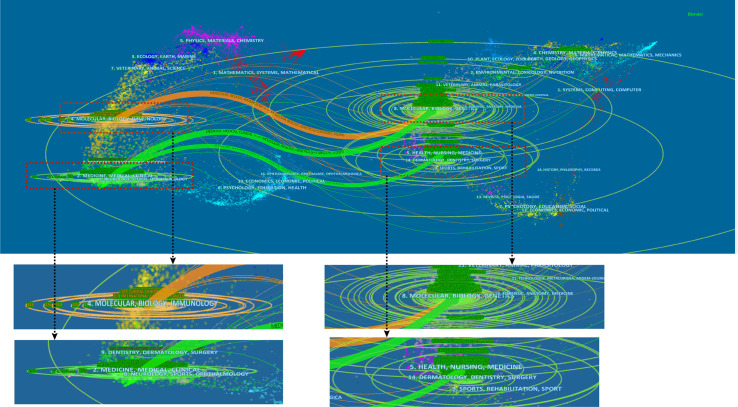
A dual-map overlay of the relevant journals generated by CiteSpace V (version5.8 R3).

**Figure 6** was a dual-map overlay of the relevant journals, which revealed the citation relationships of the journal among related fields through visualization. The labels on the map represent the field to which the journal belongs. The left side of the map represented the field of citing literature, and the right side represented the field of cited literature. Different colors represented different citation paths. The figure identified three main citation paths, including two green paths and one orange path. The orange line showed that the includes articles were mostly distributed in the fields of Molecular, Biology and Immunology, while the cited articles were mostly distributed in the fields of Molecular, Biology and Genetics. The green path indicated that the articles included in the analysis were mostly distributed in the fields of Medicine, Medical and Clinical, while the cited articles were mostly distributed in the fields of Molecular, Biology, Genetics, Health, Nursing and Medicine. The determination of citation path can represent the causal relationship of citation. The citing literature can be regarded as the applied research in this field, while the cited literature can be regarded as the research basis in this field.

### Citations and co-cited citations

**Figure 7:**
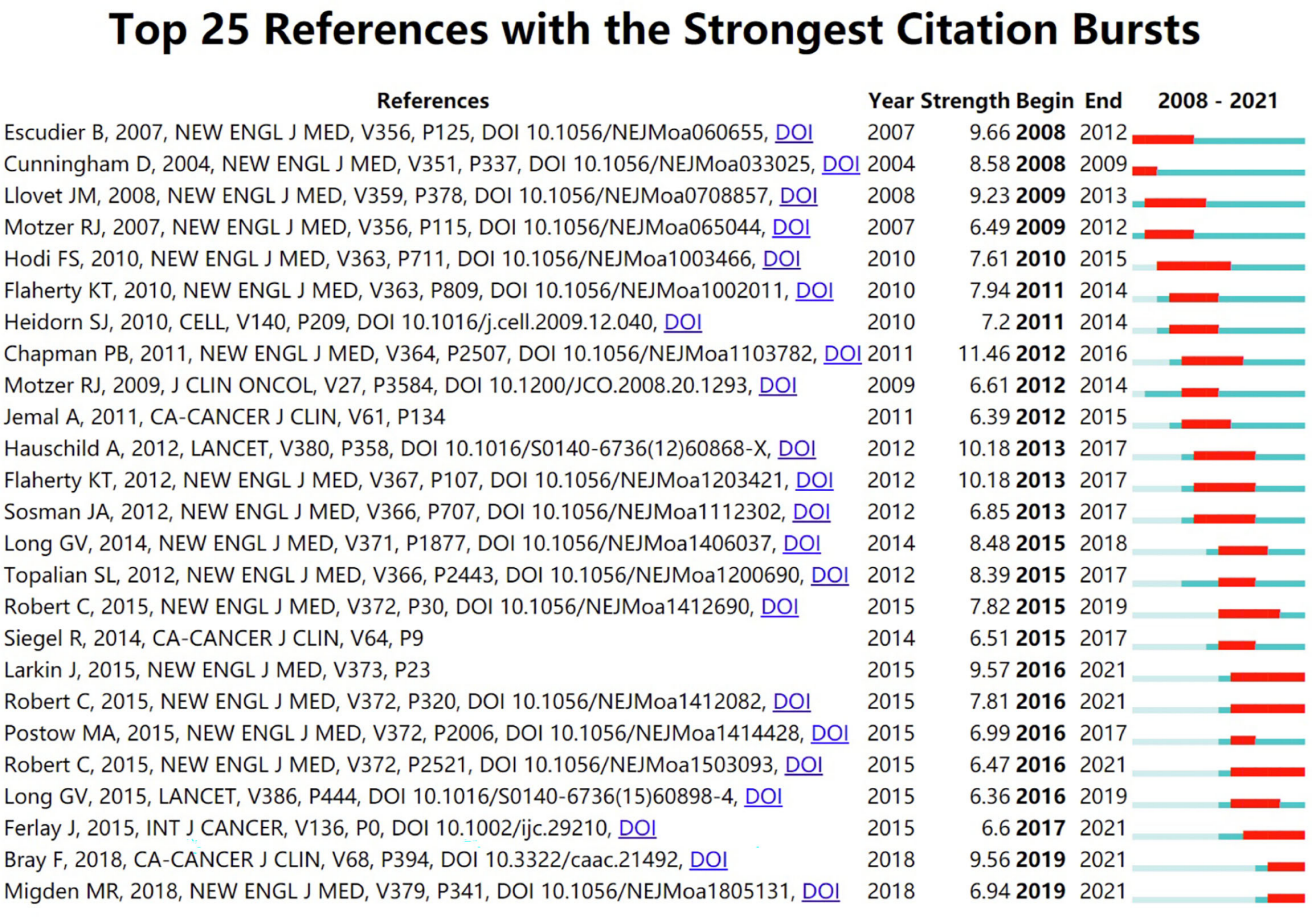
The top 25 references with the strongest citation. (The green line segment represents the time interval, and the red line segment represents the active time).

The top 10 related articles with the most citations are shown in **Table 5**. Journal of Clinical Oncology and Lancet Oncology had a huge scientific impact on researchers and scholars in this field, with more than half of the top 10 most-cited articles published in these journals. All the top 10 references were co-cited more than 320 times. The study by Michot.JM et al. (2016), published in European Journal of Cancer, was the most cited article with 1,122 times by far. CiteSpace V (version5.8 R3) was used to search for citation burstiness, and a total of 25 references with the highest citation bursts were found in **Figure 7**. References with citation bursts first appeared in 2008, while the burst originated from a paper in 2004. About 60% of the references were cited between 2012 and 2016. The most recent reference with citation burst was observed in 2019, and this burst is still ongoing.

**Table 5:** The top 10 related articles with the most citations.

Title	First author	Journal	Year	Citations	Main conclusion
Immune-related adverse events with immune checkpoint blockade: a comprehensive review	Michot.JM	European Journal of Cancer	2016	1122	They summarized the various manifestations and management of immune-related adverse events (irAEs) caused by anti-CTLA-4 antibodies and anti-PD-1/PD-L1 antibodies. Vitiligo is the most common dermatologic irAEs in melanoma patients, others including rash, erythema, Stevens-Johnson syndrome and toxic epidermal necrosis (SJS/TEN). For the treatment of grade I-II, topical corticosteroids combined with oral antipruritic drugs are effective (skin infection must be excluded before steroid applying); for grade III-IV, a skin biopsy is necessary and systemic steroids are appropriate. And pointed out that after a typical 2-4 week course of full steroid doses, steroids must be tapered over a period of at least 1 month to avoid irAEs recurrence.
Lapatinib Combined With Letrozole Versus Letrozole and Placebo As First-Line Therapy for Postmenopausal Hormone Receptor-Positive Metastatic Breast Cancer	Johnston.S	Journal of Clinical Oncology	2009	755	They found that in HR-positive, HER-2-positive metastatic breast cancer patients, letrozole combined with lapatinib can significantly improve clinical benefit rates, prolong median PFS, and reduce the risk of disease progression compared with letrozole plus placebo. But it also has a higher incidence of rashes (grade1-4, 44% vs 13%; grade3-4, 1% vs 0%).
Safety profiles of anti-CTLA-4 and anti-PD-1 antibodies alone and in combination	Boutros.C	Nature Review Clinical Oncology	2016	533	They described the adverse event profiles and mechanism of CTLA-4 and PD-1-targeted checkpoint inhibitors in melanoma patients, including rash, pruritus, enterocolitis, diarrhea, anorexia, fatigue, hypophysitis, and more. Concomitant use of both drugs was associated with a higher rate of irAEs and a wider range of adverse events. The possible association of irAEs with the clinical benefit of their treatment has not been fully elucidated. In management, grade 1-2 irAEs that do not interfere with daily live activities usually do not require dose omission or discontinuation. For patients with persistent grade 2 or more severe irAEs, symptoms should be treated with corticosteroids, and dose skipping or discontinuation is also required.
Dual-targeted therapy with trastuzumab and lapatinib in treatment-refractory, KRAS codon 12/13 wild-type, HER2-positive metastatic colorectal cancer (HERACLES): a proof-of-concept, multicentre, open-label, phase 2 trial	Sartore-Bianchi.A	Lancet Oncology	2016	481	They used trastuzumab and lapatinib dual-targeted therapy to treat 27 patients with refractory, KRAS codon 12/13 wild-type, HER2-positive metastatic colorectal cancer. The results showed that the objective response rate was 30% (CR 4%, PR 26%), 44% of patients had stable disease, 48% (13/27) of patients developed rash (1 patient had a grade 3 rash), and there were no treatment-related grade 4 and 5 adverse events.
MEK162 for patients with advanced melanoma harbouring NRAS or Val600 BRAF mutations: a non-randomised, open-label phase 2 study	Ascierto.PA	Lancet Oncology	2013	468	They evaluated the efficacy of MEK162 in patients with advanced melanoma with NRAS or Val600 BRAF mutations. Median follow-up was 3.3 months (range 0.6-8.7; IQR 2.2-5.0). No patient had a CR. Six (20%) of 30 patients with NRAS-mutant melanoma had a PR (3 confirmed) and 8 (20%) of 41 patients with BRAF-mutated melanoma had a PR (2 confirmed). Rash occurred in 6 (20%) patients with NRAS-mutant melanoma and 16 (39%) patients with BRAF-mutant melanoma. There were no deaths from treatment-related causes.
Anti-PD-1 and Anti-CTLA-4 Therapies in Cancer: Mechanisms of Action, efficacy, and Limitations	Seidel.JA	Frontiers In Oncology	2018	455	They summarized the advantages and limitations of immune checkpoint inhibitors in tumor therapy, as well as immunotherapy-related adverse event and management. Patients treated with anti-CTLA-4 had a higher incidence of side effects than those treated with anti-PD-1. Certain treatment-related autoimmune reactions such as rash and vitiligo were associated with better outcomes.
Regorafenib plus best supportive care versus placebo plus best supportive care in Asian patients with previously treated metastatic colorectal cancer (CONCUR): a randomised, double-blind, placebo-controlled, phase 3 trial	Li.J	Lancet Oncology	2015	431	They demonstrated an OS benefit from regorafenib in Asian patients with refractory metastatic colorectal cancer in a Phase 3 clinical trial. The most common grade 3 or higher adverse events associated with regorafenib were HFSR(16% in the regorafenib group), and they found the highest incidence of HFSR, rash, and fatigue within one to two weeks of initial treatment.
BRAF/NRAS Mutation Frequencies Among Primary Tumors and Metastases in Patients With Melanoma	Colombino.M	Journal Of Clinical Oncology	2012	343	They explored the relative frequency of genetic factors(BRAF/NRAS/p16CDKN2A) known to play an important role in melanoma development, and their distribution among different melanoma tissues and disease progression sites by sequencing DNA from tissue samples.
SEARCH: A Phase III, Randomized, Double-Blind, Placebo-Controlled Trial of Sorafenib Plus Erlotinib in Patients With Advanced Hepatocellular Carcinoma	Zhu AX	Journal Of Clinical Oncology	2015	335	They evaluated the efficacy of sorafenib plus erlotinib for advanced liver cancer and concluded that the combination did not improve survival in patients with advanced liver cancer. The incidence of serious adverse reactions was 58% (sorafenib/erlotinib) and 54.6%(sorafenib/placebo). The incidences of rash/descaling(51.9%vs40%),anorexia(42.5%vs37.2%)and diarrhea(76.2%vs59.4%)were higher in the sorafenib/erlotinib group, and alopecia (23.7%vs12.7%) and HFSR (47.6%vs38.1%) were higher in the sorafenib/placebo group.
Hippo-Independent Activation of YAP by the GNAQ Uveal Melanoma Oncogene through a Trio-Regulated Rho GTPase Signaling Circuitry	Feng.XD	Cancer Cell	2014	320	They found that transcriptional coactivator YAP was a suitable therapeutic target for uveal melanoma. They demonstrated that YAP activation represented a key factor in GNAQ-induced tumorigenesis and that inhibition of YAP function might represent a pharmacological intervention strategy.

### Keywords analysis of research hotspots

**Figure 8:**
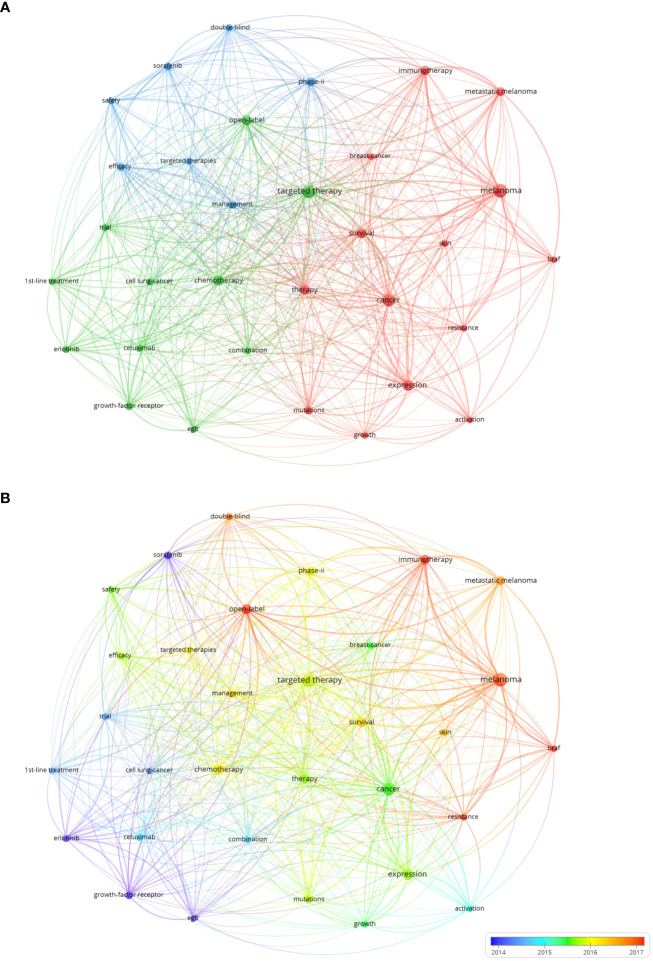
**(A)** The network visualization map of keywords generated by VOS viewer (version5.8 R3). **(B)** The visualization map of keywords over time by VOS viewer (version5.8 R3).

We extracted keywords from the titles and abstracts of 1,229 included articles. Keywords that appeared more than 50 times were used to generate a visual map through VOS viewer (version5.8 R3), which contained 32 keywords (**Figure 8A**). Cluster analysis was conducted for high-frequency keywords (occurrences greater than 50 times). There were 32 nodes and 461 links in the visualization map, and the high-frequency keywords were grouped into three clusters (Cluster 1: Red; Cluster 2: Green; Cluster 3: Blue).

**Figure 9:**
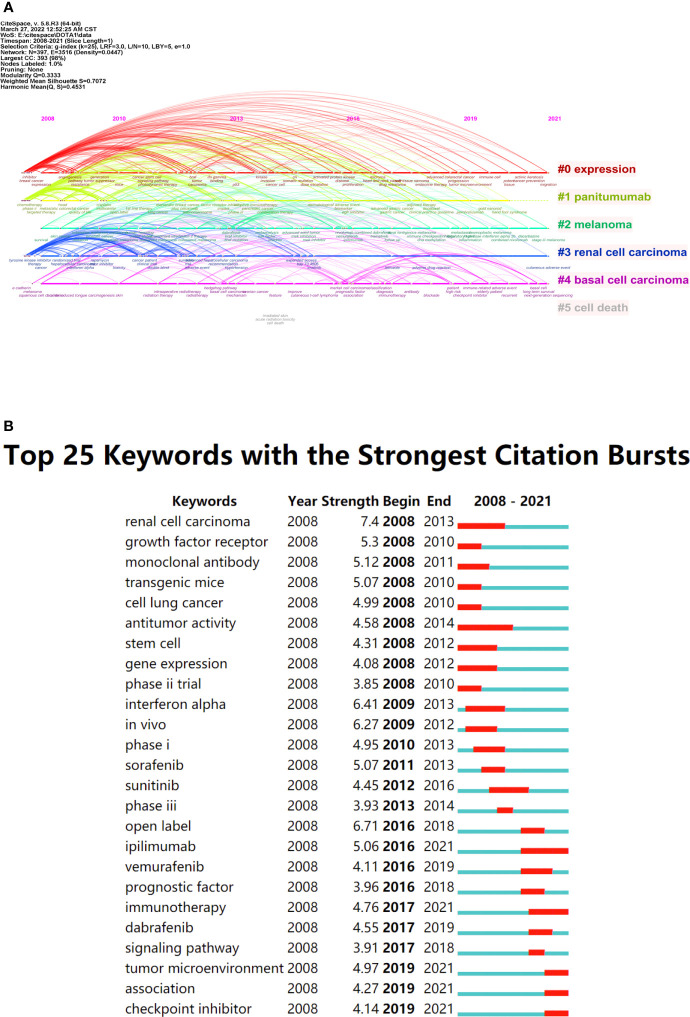
**(A)** Timeline view of keywords from publications generated by CiteSpace V (version5.8 R3). **(B)** The top 25 keywords with the strongest citation bursts of publications (The green line segment represents the time interval, and the red line segment represents the active time).

Cluster 1 was the largest, and the most frequently appeared keywords were melanoma (215 times), cancer (185 times) and expression (144 times). The main keywords of cluster 2 were targeted therapy (194 times), chemotherapy (127 times) and open label (109 times). For cluster 3, prominent keywords were phase-II (86 times), efficacy (71 times) and management (71 times). Then, we assigned keywords to the timeline, and obtained two time-varying visual maps of keywords through VOS viewer (version5.8 R3) and CiteSpace V (version5.8 R3). In **Figure 8B**, the occurrence of keywords has changed from blue to red color over time, indicating that research hotspots related to Melanoma and Immunotherapy have emerged in recent years. **Figure 9A** shows the clustering of keywords into six clusters by CiteSpace V (version5.8 R3), which reflects the hot research directions in recent years: Keratosis prevention migration, Hand-Foot Syndrome, Stage III Melanoma, Cutaneous adverse event, Next-generation Sequencing, and Cell death.

Burst keywords are another important data to reflect research hotspots and academic frontiers (**Figure 9B**). The red part indicated that the keywords show a trend of blowout at this stage. We found that there were still keywords emerging until the end of 2021, such as ipilimumab, immunotherapy, tumor microenvironment, association, checkpoint inhibitor, indicating that these research directions had been highly concerned in recent years and might become hot spots and directions of future research.

## Discussion

In this study, we examined the global scientific output associated with researches on precision cancer medicine-related rashes from 2008 to 2021 using a bibliometric analysis. As shown in **Figure 2**, the number of global publications on precision cancer medicine-related rashes increased significantly from 2008 to 2021. Before 2013, the global related publications showed a gradual growth trend. The number of publications tends to stabilize between 2013 and 2019. And in 2021, there was a substantial increase. Therefore, it is speculated that this field may enter a golden age in the next few years.

In terms of country analysis, the United States was the country with the largest output in this field, and the number of its publications is much higher than that of other countries. At the same time, the United States had the most citations, the highest H-index, and the largest TLS, suggesting that the quality and influence of articles published by the United States is higher, indicating that the United States is in a dominant position in this field, followed by Germany and France. And notably, although China ranked second in the number of publications, the citations, H-index and TLS were the bottom, pointing that Chinese scholars should pay more attention to the quality of academic articles.

Among the top 10 institutions, except for 2 laboratories from France, the remaining 8 institutions were all from the United States. This may be the reason why the United States has published the most correlational researches on precision cancer medicine-related rashes. And these results also showed that the establishment of first-scale colleges and research institutions provided a crucial foundation to promote the national academic status.

On the list of the top 10 most prolific scholars, two were from the United States, three were from China, and other five were from Canada, Italy, India, Switzerland and Germany, respectively. Lacouture ME from Memorial Sloan-Kettering Cancer Center, Robert C from Minist Environm & Lutte Changements Climat and DiGiovanni J from University of Texas Austin contributed the most publications. **Figure 5A** is an assessment of the relationship between projects by the number of co-authored documents, revealing the collaborative relationship between authors. There is a close cooperation between authors labeled with the same color. **Figure 5B** illustrates the relationship between authors based on the number of citations they have received together. Authors marked with the same color have similar research fields, and larger nodes predict dominance in this area for that author. This analysis may help new researchers to better understand existing partnerships and identify important/potential collaborators in the field. Lacouture ME and Robert C had active collaborator networks, and Robert C had the highest co-citation link strength. They had played an important leadership role in this field, more important publications related to precision cancer medicine-related rashes are more likely to be published by these authors and their teams.

In terms of top journals, those listed in **Table 4**, such as Clinical Cancer Research, Cancer Research, Oncogene, European Journal of Cancer, British Journal of Cancer may be core journals for research publications in precision cancer medicine-related rashes. This result guides scholars to submit more manuscripts to these journals. Among the top ten journals, there are two journals with IF greater than 10.0, namely Cancer Research (IF2021, 12.701) and Clinical Cancer Research (IF2021, 12.531). Five of the top ten journals, including Cancers (IF2021, 6.639), Frontiers in Oncology (IF2021, 6.244), Oncogene (IF2021, 9.867), British Journal of Cancer (IF2021, 7.640) and European Journal of Cancer (IF2021, 9.162), had an IF between 5.0 and 10.0. Overall, publishing articles related to precision cancer medicine-related rashes in high-IF journals remains a challenge.

“References with citation bursts” means that the corresponding research is frequently cited within a certain period. This indicator suggests that these publications have attracted considerable attention in the scientific community, and reflects the hot spots and dynamic changes in the field of precision cancer medicine-related rashes. The first citation burst appeared in 2008 and stemmed from the article published by Cunningham D et al. in 2004 (11), which showed that the clinical response rate of colon cancer patients with skin rash after cetuximab treatment was significantly higher than that of patients without skin reaction. This citation burst gradually drew scholars’ attention to the study on the relationship between precision cancer medicine-related rashes and clinical efficacy.

Between 2012 and 2016, about 60% of the references experienced citation bursts. Notably, the bursts of three studies are still ongoing. Among them, Larkin J et al. (12) (2015) found in the phase 3 clinical trial NCT01844505 that the incidence of rashes was 40.3% (4.8% for grade 3 or 4) in melanoma patients treated with the combination of nivolumab and ipilimumab, and the incidence of rashes in patients treated with nivolumab or ipilimumab alone was 25.9% (0.6% for grade 3 or 4) and 32.8% (1.9% for grade 3 or 4). Robert C et al. (2015) found in clinical trial NCT01721772 (13)that rashes occurred in 15.0% of melanoma patients treated with nivolumab (0.5% for grades 3 or 4, resolved rapidly with study treatment delay and/or administration of glucocorticoids), while the incidence of rashes was 2.9% in dacarbazine-treated patients with no grade 3 to 4 rashes occurred; in clinical trial NCT01866319 (14), patients with advanced melanoma treated with pembrolizumab (10 mg/kg q2w or q3w) had a longer time until the onset of the first grade 3 to 5 adverse events. The incidence of permanent discontinuation due to the treatment-related adverse events was lower in the pembrolizumab group than in the ipilimumab group. And the occurrence of rashes in each pembrolizumab group and ipilimumab group was 14.7% (q2w), 13.4% (q3w), and 14.5%, respectively. No severe grade 3-5 rashes occurred in the pembrolizumab group, compared with 0.8% in the ipilimumab group. It is noteworthy that the most recent bursts began in 2019 and remain ongoing. This is mainly related to two articles published by Bray F et al. (15) (2018) in CA Cancer J Clin and Migden MR et al. (16) (2018) in N Engl J Med.

Through the analysis of frequently occurring keywords, we can further understand the changing trends and main topics development in this field. As shown in the keyword clustering diagram in the **Figure 8A**, all the keywords of research on precision cancer medicine-related rashes could be divided into 3 categories. Cluster 1 mainly focused on the therapeutic targets of tumors, and the prominent keywords were melanoma, cancer, and expression. Cluster 2 was primarily about clinical application of targeted therapy, and the main keywords were targeted therapy, chemotherapy and open label. Cluster 3 mainly focused on the efficacy of precision cancer medicine including targeted and immunotherapy, and the management of related adverse events. The primary keywords were phase-II, efficacy and management. As a rapidly developing field, precision cancer medicine started from the basic research of tumorigenesis mechanism, and the development and clinical application of the drug were correspondingly carried out, and the related adverse events also followed. Therefore, when researching the mechanism of precision cancer medicine-related rashes in the future, we should also focus on exploring how to effectively manage it without reducing the anti-tumor intensity or terminating anti-tumor therapy.

According to the timeline view and the top 25 most cited keywords in publications exported by CiteSpace V (version5.8 R3), it can be seen that in the past few years, there has been a certain research foundation for targeted therapy-related cutaneous adverse events (AEs): Hand-foot-skin reaction (HFSR), in particular, is one of the most common targeted therapy-induced AEs, will lead to treatment interruption or failure. Some achievements have been made in the diagnosis, prevention, evaluation, management and possible mechanism of HFSR (17–22). At the same time, four potential research hotspots and frontiers can also be predicted, as follows: “checkpoint inhibitors”, “cutaneous adverse events”, “association” and “tumor microenvironment”.

(1) Checkpoint inhibitors: Since the discovery of immune antitumor responses was first published about a century ago (23), the research on tumor immunotherapy has grown step by step. Immunotherapy has gradually become a viable treatment option for a variety of tumors over the past 30 years. Anti-cytotoxic T lymphocyte-associated protein 4 (CTLA-4) was approved in 2011 for the treatment of advanced melanoma, and other immune checkpoint inhibitors (ICIs) were quickly approved by the Food and Drug Administration (24). Today, the profound and long-lasting antitumor effects of immune checkpoint inhibitors have revolutionized oncology and altered the prognosis of many cancers (25–28).

Notwithstanding, resistance to ICIs limits the number of patients who can achieve a lasting response. With the study of the mechanisms of tumor immune evasion after ICIs treatment, we have improved our understanding of the basis for efficacy and resistance, and we are achieving even more impressive success through different combinatorial strategies, including combinations of different immune checkpoint inhibitors, or combination with targeted therapies, chemotherapy and so on. For example, the 5-year OS of the combination of PD-1 inhibitor nivolumab and CTLA-4 inhibitor ipilimumab was unprecedented higher than 50% (29). And OS, progression-free survival (PFS), and objective response rate (ORR) were significantly improved in patients receiving the combination of ICIs and anti-VEGF, although with the expense of increased AEs (27). Published trials of combinations of BRAF/MEK inhibitors and ICIs have produced conflicting results in PFS, and the optimal sequence of BRAF/MEK inhibitors and ICIs is still pending on reliable clinical data (30, 31). In addition, novel combinations of immune checkpoint inhibitors (ICIs), such as cytokines, oncolytic viruses, TLR9 agonists, HDAC inhibitors, DNMT inhibitors, etc, are under investigation (32–35).

On the road to treat, these new methods and the individualized application of combination therapy have important research significance to the best benefit-risk ratio in clinical practice, which can improve the long-term survival rate of cancer patients further.

(2) Cutaneous adverse events: Along with the use of various targeted therapies and immunotherapies, new adverse events have emerged, including cutaneous toxicities (up to 30-60%), which range from limited morbilliform eruptions, maculopapular rash to diffuse bullous rash and even dermatological emergencies with high mortality rates such as Stevens-Johnson Syndrome (SJS), Toxic Epidermal Necrolysis, and drug reaction with eosinophilia and systemic symptoms (DRESS) (36). These cutaneous adverse events will not only greatly impact the patient’s quality of life, they can also lead to cessation or discontinuation of treatment, and affect patient survival (37).

The study found that compared with chemotherapy and targeted therapy, patients treated with ICIs had an increased risk of any related adverse events (RR, 2.65; 95% CI, 1.84-3.83; *P* < 0.00001), including rash (1.58; 0.98-2.54) and other cutaneous adverse events (38). Compared with PD-1 ICIs (34-42%) or PD-L1 ICIs monoclonal treatment (20%), CTLA-4 ICIs monoclonal treatment was associated with a higher incidence of cutaneous adverse events (44-59%), and the incidence of combination therapy was the highest (59-72%) (39). And treatment adjustments due to cutaneous adverse events were more common in patients treated with targeted therapies and ICIs than with chemotherapy. The cutaneous adverse events that led to most treatment modifications was skin rash (40). To date, several guidelines for the management of immunotherapy-associated rashes have developed, mainly based on case reports, series, experience, and expert consensus. However, the effectiveness of these treatments is rarely reported (41, 42).

The occurrence and treatment of precision cancer medicine-related rashes complicate antitumor therapy, and therapeutic measures must be taken as soon as possible to reduce their severity and duration. Therefore, accurate diagnosis and effective management are important bottlenecks to be solved.

(3) Association: In recent years, more and more studies have shown that the occurrence of irAEs means that patients benefit from immunotherapy, including non-small cell lung cancer (43, 44), renal cell carcinoma (45), cutaneous melanoma (46), etc. Johnston.S et al. (47)found that in HR-positive, HER-2-positive metastatic breast cancer patients, letrozole in combination with lapatinib significantly improved clinical benefit rates, prolonged median PFS, and reduced risk of disease progression compared with letrozole in combination with placebo, while also resulting in a higher incidence of rash (grades 1-4, 44% vs. 13%; grades 3-4, 1% vs. 0%). In patients with different tumors such as melanoma and NSCLC using PD-1 immune checkpoint inhibitors, teams such as Freeman-Keller M (46), Akano Y (48), Quach HT (49), Lee YJ (50), Aso M (51), Bottlaender L (52) have observed that ORR, PFS and OS of patients who developed skin reactions such as rash were significantly better. A systematic review of 137 immunotherapy studies showed that the occurrence of vitiligo did not have an advantage in long-term PFS or OS, while the occurrence of remaining irAEs proved to be a survival advantage in long-term follow-up (53, 54), mainly in terms of prolonged PFS and OS and significant improvement in ORR. There are no articles reporting that patients who developed rash after receiving precision cancer medicine had worse disease remission rates currently. The mechanism of precision cancer medicine-related rashes has not been fully elucidated, but its clinical significance has attracted extensive attention. At present, most retrospective studies have confirmed this conclusion, which needs to be further verified in larger prospective clinical studies.

(4) Tumor microenvironment: ICIs are monoclonal antibodies that block receptors, which lead to the activation of immune cells in the tumor microenvironment (55, 56), and the benefits of cancer therapy are accompanied by autoimmune side effects known as irAEs. IrAEs caused by ICIs are thought to occur through several immunologic pathways. In the physiologic state, CTLA-4 is involved in thymic maturation of T cells and downregulating T cell activation, while the PD-1 pathway is involved in the induction and maintenance of peripheral tolerance against self-reactive T cells. When these pathways are pharmacologically blocked, T cell responses are promoted, leading to both antitumor responses and the proliferation of self-reactive T cells with resultant autoimmunity (39). These reactions typically affect the skin, colon, liver, lungs, endocrine organs, and joints (56, 57). Another idea is that cross-reactive T cells (T cells that bind to tumor and irAEs target tissues) may play a role, mechanism involves cross reactivity between antigens on the target tumor cells and self-antigens on normal host tissues. Vitiligo in particular has been linked to cross-reactivity between melanoma-associated antigens and melanocytes, both of which may become targets of the ICI associated immune response (58, 59). Bullous dermatitis is a type of immune-associated rash, and a study (60) found that basement membrane protein BP180, which targets newborn bullous dermatitis, may mediate this reaction. In addition, cytokines and chemokines may also be important mediators in the pathogenesis of irAEs (61), but there is a lack of high-level evidence to support this.

## Strengths and limitations

Our study is the first time to use bibliometric analysis and visualization tools to analyze global trends of precision cancer medicine-related rashes, which systematically demonstrates the evolution, status, and frontiers of related researches. The limitations of this study are as follows: first, we only retrieved and collected literature data from the WOSCC database, which may miss important studies in PubMed, Embase and other databases. Second, the study only included English literature, and important researches published in other languages may be ignored. Finally, only the journal’s impact factor and quartile in category were evaluated, and the quality of the articles included in the study was not assessed.

## Conclusion

To conclude, the study of precision cancer medicine-related rashes is in a developmental stage, and the number of relevant publications will increase rapidly. The United States has the largest quantity of studies on this topic and the highest quality and impact of its articles, is playing a guiding role in this field. At present, the research focus is gradually shifting to tumor immunotherapy, “checkpoint inhibitors”, “skin adverse events”, “association” and “tumor microenvironment” may become the future research hotspots.

## Data availability statement

The original contributions presented in the study are included in the article/supplementary material, further inquiries can be directed to the corresponding author.

## Author contributions

FZ contributed to conceptualization, visualization and writing-original draft, review and editing. RY contributed to conceptualization, visualization and writing-original draft, review and editing.SC contributed to conceptualization, funding acquisition and writing-original draft, review and editing. SZ contributed to writing-review and editing. LS contributed to writing- review and editing. ZX contributed to writing-review and editing. YZ contributed to writing-review and editing. SD contributed to writing-review and editing. GZ contributed to writing-review and editing. QS contributed to supervision, funding acquisition and writing-review and editing. All authors contributed to the article and approved the submitted version.

## Funding

This work was supported by the following research grants from clinical (gastric cancer) cooperation pilot project of Chinese and Western Medicine for Major; and Difficult Diseases and Zhejiang Traditional Chinese Medicine of Science and Technology Program (No. 2020ZA053).

## Conflict of interest

The authors declare that the research was conducted in the absence of any commercial or financial relationships that could be construed as a potential conflict of interest.

## Publisher’s note

All claims expressed in this article are solely those of the authors and do not necessarily represent those of their affiliated organizations, or those of the publisher, the editors and the reviewers. Any product that may be evaluated in this article, or claim that may be made by its manufacturer, is not guaranteed or endorsed by the publisher.
